# Photobleaching-based autofluorescence mapping reveals distinct patterns in sporadic basal cell carcinoma, nevoid basal cell carcinoma syndrome-associated basal cell carcinoma and squamous cell carcinoma

**DOI:** 10.3389/fmed.2026.1773895

**Published:** 2026-04-14

**Authors:** Emilija V. Plorina, Ilze Lihacova, Mehdi Boostani, Szabolcs Bozsányi, Norbert Kiss, Dmitrijs Bliznuks, Marta Skrastina, Alexey Lihachev

**Affiliations:** 1 Faculty of Science and Technology, Institute of Atomic Physics and Spectroscopy, University of Latvia, Riga, Latvia; 2 Department of Dermatology, Roswell Park Comprehensive Cancer Center, Buffalo, NY, United States; 3 Department of Dermatology, Venereology and Dermatooncology, Semmelweis University, Budapest, Hungary; 4 Institute of Applied Computer Systems, Riga Technical University, Riga, Latvia

**Keywords:** autofluorescence, NBCCS, non-invasive skin imaging, photobleaching imaging, skin cancer

## Abstract

**Introduction:**

Autofluorescence (AF) imaging is a promising non-invasive tool for skin cancer diagnostics. However, AF intensity alone often fails to reliably delineate tumor boundaries due to strong influence from melanin, keratin, vasculature, and surface scattering. AF photobleaching kinetics provide an additional functional contrast linked to endogenous fluorophore content and environment microstructure, but their diagnostic potential in clinical skin cancer imaging remains underexplored.

**Methods:**

To investigate the photobleaching phenomenon, we analyzed AF and photobleaching behavior in 84 histologically confirmed lesions, including 53 sporadic basal cell carcinomas (BCC), 25 BCCs associated with nevoid basal cell carcinoma syndrome (NBCCS, also known as Gorlin syndrome), and 6 squamous cell carcinomas (SCC). Continuous narrow band LED 405 nm excitation for 20 s enabled pixel-wise fitting of exponential decay curves, generating photobleaching parameter maps describing bleaching amplitude (A), decay constant (τ), and residual component (C).

**Results:**

Across all tumor types, lesions consistently exhibited lower initial AF, reduced photobleaching amplitude, and faster τ compared to surrounding skin. A key qualitative finding was a systematic mismatch between lesion boundaries seen in AF intensity versus boundaries revealed by A and τ maps, suggesting that photobleaching parameters capture deeper or structurally distinct tissue alterations not visible in steady-state AF emission imaging. NBCCS-associated BCCs showed more homogeneous A/τ patterns than sporadic tumors, while SCC lesions demonstrated coarse, irregular τ distributions distinct from BCC.

**Discussion:**

These observations indicate that photobleaching kinetics provide biologically meaningful contrast and may improve non-invasive tumor characterization, with potential relevance for early diagnosis and surgical margin assessment.

## 1 Introduction

Basal cell carcinoma (BCC) is the most common human malignancy and the most frequent form of non-melanoma skin cancer among individuals with fair skin, with incidence rates rising globally by 2–10% per year (1–3). Established risk factors include cumulative ultraviolet (UV) exposure, older age, lighter skin phototypes, and a prior history of skin cancer (4, 5). Although the molecular mechanisms underlying BCC development are multifactorial, UV-induced genetic damage, particularly in individuals with intrinsically lower epidermal photoprotection, plays a central role in tumor initiation and progression (6). Nevoid basal cell carcinoma syndrome (NBCCS), also known as Gorlin syndrome is a rare autosomal dominant disorder caused by mutations in *PTCH1*, *PTCH2*, or *SUFU* key regulators of the Sonic Hedgehog pathway (6–11). NBCCS is characterized by a markedly increased lifetime tumor burden, with patients frequently developing dozens to hundreds of BCCs, often beginning in early adulthood. The median age of onset in NBCCS is approximately 25 years, compared to 65 years in sporadic BCC (12, 13). Moreover, NBCCS lesions tend to be biologically and morphologically homogeneous, whereas sporadic BCCs typically display greater lesion-to-lesion variability. Given the high cumulative morbidity, NBCCS patients often undergo repeated minimally invasive treatments such as cryotherapy, photodynamic therapy, or topical immunomodulators rather than excisional procedures, which may lead to scarring (14, 15). These constraints emphasize the need for reliable, non-invasive imaging techniques capable of distinguishing tumor from surrounding skin and defining lesion boundaries with minimal patient discomfort. AF imaging has been explored as a diagnostic tool for skin tumors due to the altered distribution of endogenous fluorophores such as lipofuscin, tryptophan, NAD(P)H, and structural proteins in malignant tissue (16–20). However, AF intensity is highly susceptible to surface absorption, melanin content, vascular shadows, and illumination homogeneity, often producing ambiguous or misleading lesion boundaries. AF photobleaching (PB), in contrast, provides a dynamic contrast mechanism: the temporal decay of fluorescence under continuous excitation reflects the biochemical environment, fluorophore composition, and microstructural organization of tissue. Theoretical models suggest that PB may arise from irreversible fluorophore photodecomposition or light-induced transitions to non-fluorescent states (17). Previous studies of AF photobleaching in skin tumors have been limited in scope, typically comparing mean intensity decay curves without assessing spatial heterogeneity. In contrast, the present work examines pixel-wise maps of photobleaching parameters: bleaching amplitude (A), decay constant (*τ*), and residual component (C) obtained from exponential fitting of continuous AF imaging under 405 nm excitation. This approach allows visualization of spatially resolved photobleaching behavior across lesions. Here, we investigated 53 sporadic BCCs, 25 NBCCS-associated BCCs, and 6 squamous cell carcinomas (SCCs) to determine whether AF photobleaching maps reveal qualitative differences between tumor types and whether they provide lesion boundaries that differ from or improve upon static AF intensity. Special attention was given to (i) the consistency of photobleaching signatures across the visible area of the lesions, (ii) differences in intratumoral heterogeneity between NBCCS-associated and sporadic BCC, and (iii) the frequently observed boundary mismatch between AF and PB derived lesion contours. Understanding these qualitative patterns may support the development of non-invasive, functionally informative imaging methods for early tumor detection and surgical margin planning.

## 2 Materials and methods

AF imaging data were collected from patients diagnosed with BCC at the Department of Dermatology, Venereology and Dermato-oncology, Semmelweis University, Budapest, Hungary, under the supervision of a consultant dermatologist. In total, 84 histologically verified lesions were included in the present analysis: 53 sporadic BCC, 25 BCCs associated with NBCCS, and 6 SCC. AF imaging was performed for all lesions prior to surgical excision, and all diagnoses were confirmed by an expert dermatopathologist. The NBCCS group consisted of both male and female patients carrying clinical and/or genetic hallmarks of the syndrome, while the sporadic BCC and SCC groups included patients without features of hereditary tumor predisposition.

### 2.1 Patient selection, inclusion, and exclusion criteria

The inclusion criteria required written informed consent, histopathological confirmation of BCC or SCC by a board-certified dermatopathologist, and the presence of a lesion in a location where stable, reproducible AF imaging could be performed. In contrast, the exclusion criteria encompassed any condition that could compromise image acquisition, including active bleeding or ulceration; prior interventions such as biopsy, surgery, topical therapies, or Hedgehog pathway inhibitors; and tumors situated on anatomically challenging or highly curved regions, such as the eyelids, eyebrows, ears, or peri-nasal folds, where the device could not be positioned adequately on the skin surface, rendering AF imaging infeasible.

### 2.2 Ethics approval

The study was conducted according to the guidelines of the Declaration of Helsinki and approved by the Institutional Ethics Committee of Semmelweis University (SE RKEB no. 16/2022).

### 2.3 Imaging system

AF and AF PB measurements were obtained using a custom-built imaging device equipped with a 405 nm LED source (irradiance ≈ 7 mW/cm^2^) for cutaneous AF excitation. A 515 nm long-pass optical filter was placed in front of the detector to block reflected excitation light, and cross-polarizing filters were incorporated to minimize surface glare. Images were captured in RGB format by a CMOS sensor and transferred to a computer for further analysis. The structural design and data acquisition architecture of the device have been described previously by Osipovs et al. (21).

### 2.4 Image acquisition

AF imaging was performed by acquiring 8-bit RGB images with a spatial resolution of 466 × 448 pixels at a frame rate of 1 frame per second over a 20 s acquisition period. During image acquisition, continuous narrow-band LED illumination at 405 nm was applied to induce photobleaching of endogenous skin fluorophores. This acquisition protocol resulted in a temporal autofluorescence decay curve for each pixel within the field of view (approximately 24 × 25 mm). To reduce the influence of minor patient motion during the measurement, the acquired image sequences were subjected to frame-to-frame stabilization prior to further processing.

### 2.5 Photobleaching model and parameter extraction

Instead of relying solely on intensity subtraction between the first and last frames, the present study employed pixel-wise exponential curve fitting to characterize fluorescence decay dynamics. Previous studies have applied either single-exponential decay, double-exponential decay or non-exponential decay functions (22–24) for the PB curve depending on the specifics of the method of data acquisition. By testing linear, single and double-exponential models we found that a single-exponential model yielded the least errors during regression analysis. Linear and double-exponential decay model parameter maps acquired during model testing are omitted from this paper. For each pixel, the temporal intensity profile I(T) was fitted using a standard single-exponential photobleaching model described by Equation 1:


$$I(t)=A\cdot e^{-\frac{t}{\tau }}+C$$ (1)

Variable t represents time of measurement, I(t) represents AF intensity at time t, while parameters A, *τ*, and C describe the exponential decay of AF intensity over time t which can also be termed as amplitude, decay constant and residual component, respectively. Fitted parameters were assembled into spatially resolved A, *τ*, and C maps, which were analyzed alongside the initial AF intensity image (*t* = 0 s). This produced a set of four functional images per lesion: AF, A, τ, and C.

### 2.6 Data processing and qualitative comparison

AF intensity I(t) is extracted from the G channel of the RGB image as it contains the majority of the autofluorescence signal. All processing algorithms were created using Python 3.11 and R 4.5.2. Pixel fitting was done using the SciPy library function scipy.optimize.least_squares which is used for nonlinear problem solving. The function was applied on each individual stack of pixels with input being time of measurement and value of pixel at the time of measurement. For pixels that could not be fitted, the output values were set to 0, while for pixels that were poorly fitted the algorithm output infinite or very large parameter values in addition to large error values, which were excluded from further analysis.

Segmentation of intensity and parameter maps was done using the Scikit-image library from which the simple linear iterative clustering (SLIC) segmentation method was the primary segmentation algorithm used, based on a k-means clustering approach (25). The SLIC segmentation could generate well demarcated superpixels (clusters of pixels with similar values) that could be used to mark the shape of the lesion according to the parameter value used. The mean parameters of a superpixel that deviated sharply from surrounding skin were marked as core lesion superpixels, while superpixels with deviated values around the core superpixels were designated as junction superpixels. Automatic segmentation algorithms of these areas were tested but were deemed unreliable as parameter values and lesion presentation varied too much between patients.

Statistical calculations and statistical figure production were done using the R programming language with libraries car, rstatix, ggplot2 and built-in functions. Lesion group homogeneity was checked using Levene’s test. Difference between independent groups was tested with Kruskal-Wallis, while pairwise comparison between groups was done using Dunn’s test with Bonferroni adjustment.

## 3 Results

### 3.1 AF photobleaching kinetics of non-melanoma skin cancer cases included in the study

**Figure 1:**
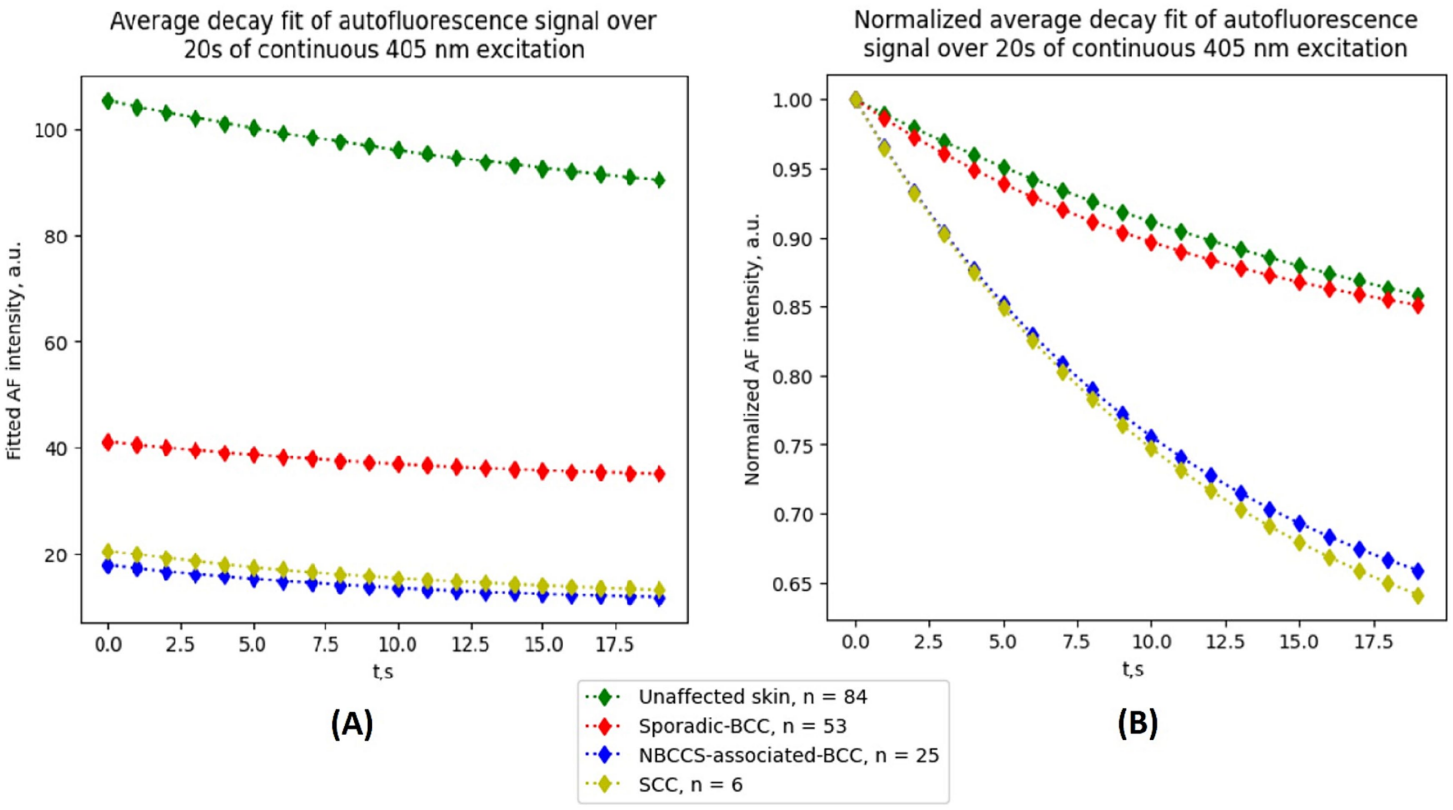
Photobleaching behavior of autofluorescence of sporadic BCC, NBCCS-associated BCC, SCC, and unaffected surrounding skin. **(A)** Average intensity decay curves calculated from a 20 s continuous 405 nm excitation period **(B)** Normalized autofluorescence (AF) photobleaching curves from the same regions for emphasis of the decay curve shape; for normalization the initial value of the decay curve is used.

In Figure 1, the PB kinetics of AF decay during 20 s of continuous excitation with a narrow-band 405 nm LED are shown. The kinetics were obtained for different types of non-melanoma skin cancer as well as healthy skin, namely sporadic BCC, nevoid BCC, and SCC. Each kinetic curve represents the average AF intensity change over the 20 s acquisition period, calculated from three representative cancer cases and four healthy skin measurements. The results presented in the figure demonstrate the absolute decay values (right panel) as well as the normalized decay curves (left panel). As can be seen, all malignant cases exhibit substantially lower AF intensity and a more pronounced photobleaching effect compared to healthy skin. However, for a more accurate comparison, normalized data should be considered, where the characteristic decay shape over the first 20 s becomes more evident. All three tumor types, as well as healthy skin, can be approximated using a single-exponential expression, allowing calculation of the exponential parameters a, *τ*, and c. Each tumor type shows a distinct combination of these parameters. In particular, sporadic BCC is characterized by a moderately accelerated decay compared to healthy skin, whereas NBCCS-associated BCC exhibits a more pronounced reduction in normalized AF signal over time, indicating faster photobleaching kinetics. Relative to healthy skin, NBCCS-associated BCC still demonstrates lower baseline AF intensity, but its temporal decay behavior clearly diverges in magnitude and shape. SCC likewise shows reduced initial AF intensity and the steepest decay profile among the evaluated groups, reflecting a distinct combination of exponential parameters compared with both BCC subtypes and healthy tissue.

**Figure 2:**
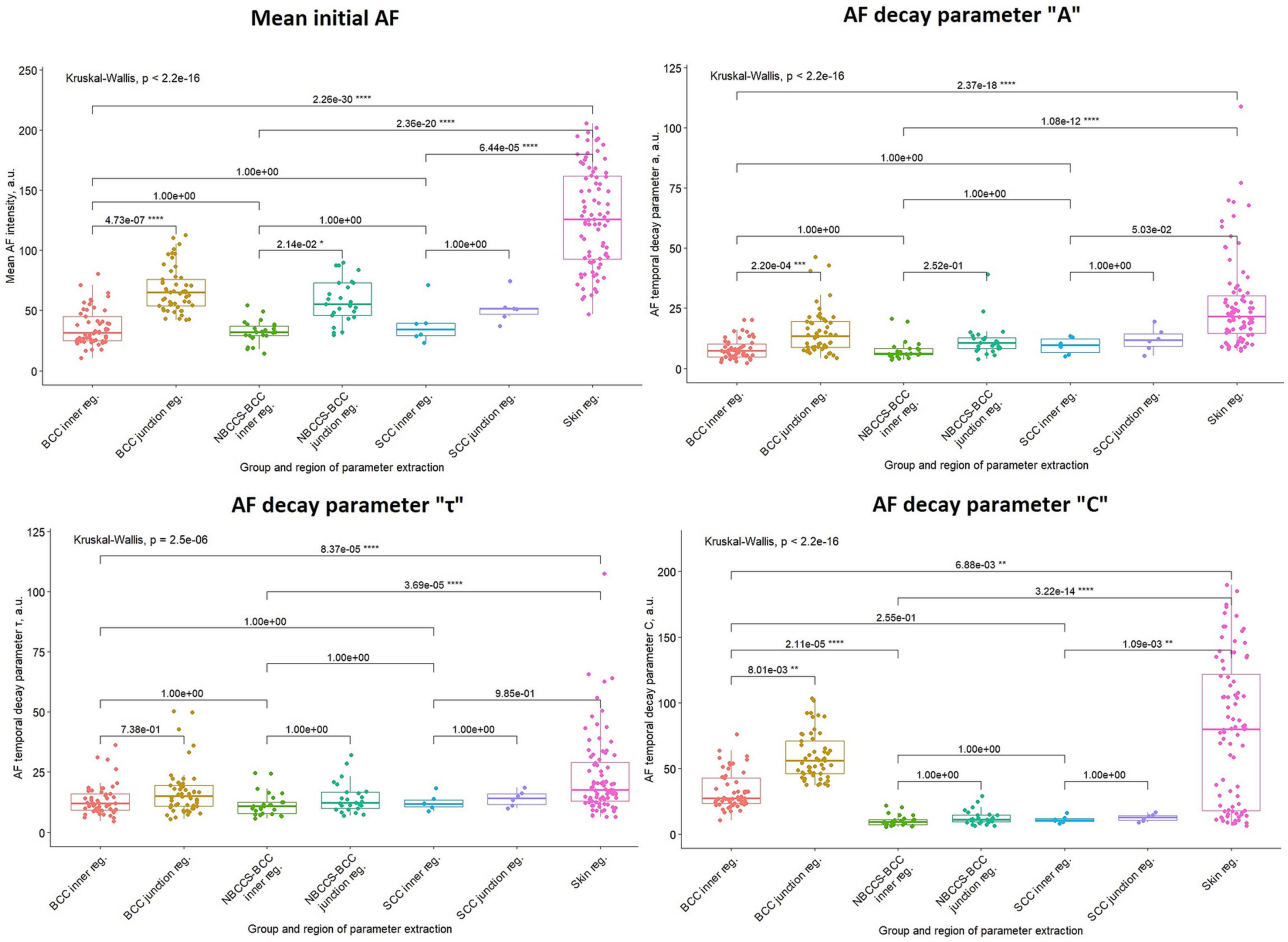
Comparison of the means of BCC, NBCCS-associated-BCC, SCC lesion groups inner, junction regions, and skin region across parameters mean initial AF intensity and temporal decay parameters “A,” “*τ*,” and “C.” Results of Kruskal-Wallis and Dunn-Bonferroni post-hoc pairwise comparison tests are shown. An asterisk “*” indicates a statistically significant result in the pairwise comparison with the number of asterisks indicating a higher level of significance.

Statistical comparisons across lesion types and anatomical regions were performed using the Kruskal–Wallis test with Dunn–Bonferroni post-hoc test (Figure 2). Significant differences between tumor inner regions and surrounding skin were observed for sporadic BCC and NBCCS-associated BCC across all evaluated parameters. In SCC, significant tumor–skin differences were detected for initial AF intensity and the residual photobleaching component C. Within sporadic BCC, significant differences between inner and junction regions were identified for initial AF, A, and C, supporting the presence of intralesional heterogeneity in both steady-state and dynamic parameters. In contrast, NBCCS-associated BCC demonstrated fewer region-specific differences, with significance primarily observed for initial AF. Direct comparison of inner tumor regions between sporadic and NBCCS-associated BCC revealed a significant difference in parameter C. Among all evaluated metrics, the residual component C demonstrated the most consistent group-level discrimination. It significantly differentiated tumor from surrounding skin across lesion types and additionally distinguished sporadic BCC from NBCCS-associated BCC. Compared to A and *τ*, which exhibited greater overlap between groups, parameter C provided the strongest and most reproducible cohort-level contrast.

### 3.2 AF photobleaching images

Across all examined non-melanoma skin cancer cases, AF imaging consistently revealed reduced fluorescence intensity within the lesions compared with surrounding skin. When fitted with a single-exponential model, the resulting parameter maps (A, *τ*, C) provided additional visual contrast and highlighted tumor-specific photobleaching behavior. Although all tumor types demonstrated lower AF and measurable alterations in photobleaching kinetics, their profiles differed: sporadic BCC typically showed reduced A and faster τ, nevoid BCC exhibited kinetics closer to normal skin despite similarly low AF intensity, and SCC presented the strongest heterogeneity with distinct parameter distributions. These general trends are illustrated below through representative cases from each diagnostic group.

### 3.3 Selected cases of sporadic BCC

Across the three representative cases of sporadic BCC, the AF intensity images consistently showed areas of lower autofluorescence compared to the surrounding healthy skin, which delineates the lesion but does not fully describe its internal structure. In contrast, the photobleaching-derived parameter maps (A, *τ*, C) revealed additional spatial features not visible in the AF images. The A-maps demonstrated heterogeneous intralesional patterns, with several regions showing locally increased photobleaching amplitude. The *τ*-maps showed the most notable differences, as the areas of faster decay did not strictly follow the AF-defined boundary and frequently extended into the adjacent skin. The C-maps also displayed peripheral gradients, indicating gradual changes in residual fluorescence outside the region of lowest AF intensity.

In the presented sporadic BCC cases, AF intensity images consistently show reduced AF signal within the lesion compared with the surrounding healthy skin, providing an initial visual delineation of the tumor area. Quantitative values extracted from the marked regions indicate that mean AF intensity within the lesion is substantially lower than in adjacent skin. Analysis of the PB-derived parameter maps reveals additional spatial information: regions characterized by altered photobleaching kinetics, most prominently reflected in the τ maps, frequently extend beyond the AF-defined lesion boundaries. The areas exhibiting modified τ behavior are consistently larger than the AF-defined lesion areas, as illustrated by the annotated region sizes on the maps. Mean values of the PB amplitude parameter A are reduced within the tumor, while *τ* values indicate faster decay kinetics compared with surrounding tissue. The C maps show more gradual spatial transitions across the lesion–skin interface. Although the numerical values are presented descriptively and are not intended for statistical comparison, they provide quantitative context that supports the visual observation of boundary mismatch and illustrates the magnitude of spatial differences between steady-state AF intensity and PB-derived maps. Together, these findings indicate that PB mapping captures tissue alterations extending beyond the boundaries suggested by AF imaging alone and may serve as a basis for future region-of-interest–based analysis, correlation with histopathology, and assessment of potential clinical relevance.

### 3.4 Selected cases of NBCCS-associated BCC

**Figure 3:**
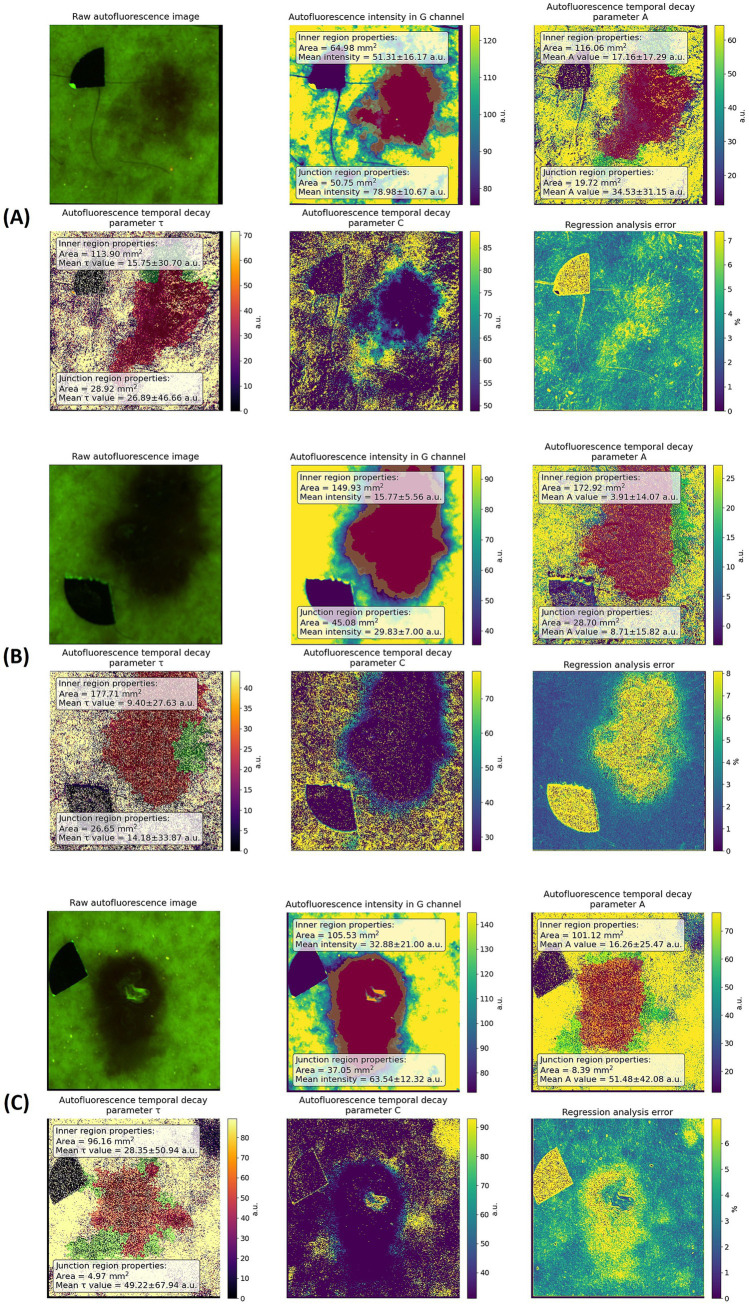
Representative examples of sporadic BCC on a 75-year-old male patient **(A)**, 53-year-old male patient **(B)** and 49-year-old female patient **(C)**. Unprocessed AF image (upper-left), AF intensity image with inner and junction region values (upper-middle), corresponding PB derived parameter maps of bleaching amplitude A with inner and junction region values (upper-right), decay constant τ with inner and junction region values (lower-left), residual component C (lower-middle), regression error (bottom-right).

In the nevoid BCC cases, AF intensity was consistently lower inside the lesion compared with the surrounding skin, but the PB-derived parameter maps revealed a distinctly different pattern from sporadic BCC. While the A-map still showed intralesional heterogeneity and reduced amplitude values, the *τ*-map demonstrated kinetics that were nearly indistinguishable from those of the surrounding healthy skin (Figure 3).

**Figure 4:**
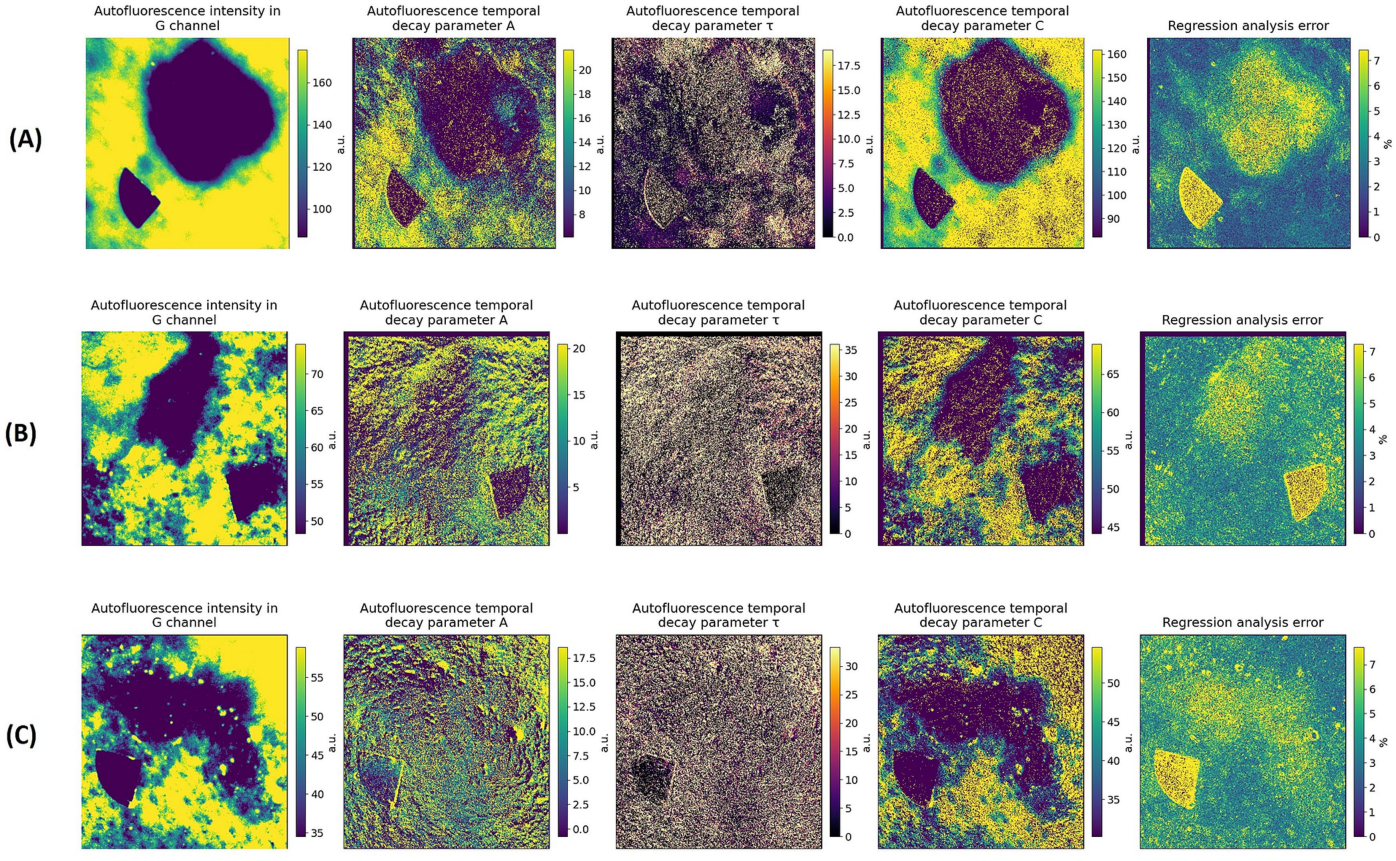
Representative examples of NBCCS-associated BCC on a 45-year-old female patient **(A,B)**, and 33-year-old female patient **(C)**. Unprocessed AF image, AF intensity image, corresponding PB derived parameter maps of bleaching amplitude A, decay constant τ, residual component C, regression error.

**Figure 5:**
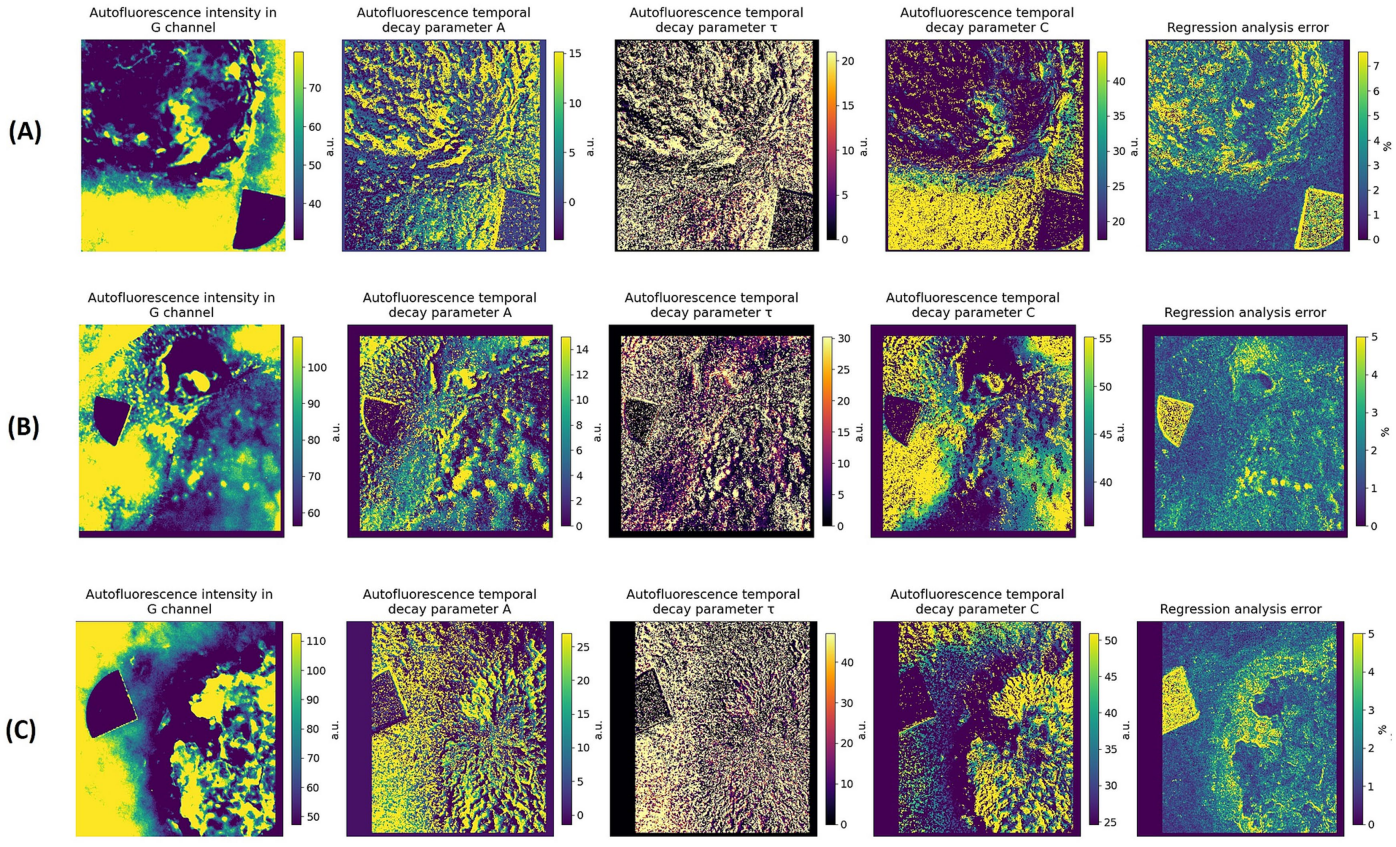
Representative examples of SCC on a 71-year-old female patient **(A)**, 74-year-old female patient **(B)**, and 71-year-old male patient **(C)**. Unprocessed AF image, AF intensity image, corresponding PB derived parameter maps of bleaching amplitude A, decay constant τ, residual component C, regression error.

Figure 4 presents representative NBCCS-associated BCC cases visualized by AF PB parameter mapping. In these lesions, AF intensity images show a clear reduction of AF within the tumor compared with surrounding healthy skin, indicating a detectable contrast at the level of steady-state intensity. However, in contrast to sporadic BCC, the photobleaching-derived parameter maps reveal markedly weaker PB contrast between the lesion and adjacent tissue. In particular, the *τ* maps show values within the nevoid BCC that are largely comparable to those of surrounding skin, consistent with the PB kinetics presented in Figure 1, where nevoid BCC exhibits decay curves closely overlapping with healthy skin despite lower baseline AF intensity. The A maps indicate reduced PB amplitude within the lesion, while the C maps display relatively smooth spatial transitions without pronounced peritumoral extension. Together, these observations indicate that although nevoid BCC is characterized by reduced AF intensity, its PB kinetics remain similar to those of healthy skin, suggesting a lower degree of kinetic alteration compared with sporadic BCC (Figure 5).

### 3.5 Selected cases of SCC

In the SCC cases, the AF images show markedly reduced intensity within the tumor region compared with the surrounding skin, similar to BCC but with a more irregular and fragmented morphology. The PB parameter maps, however, reveal a substantially more heterogeneous pattern than in either sporadic or nevoid BCC. The A-maps demonstrate pronounced spatial fluctuations with sharp local maxima and minima, while the *τ*-maps highlight zones of accelerated decay that often extend beyond the AF-defined boundaries.

This indicates deeper or more abrupt biochemical changes within the tissue architecture. The C-maps likewise show steep gradients, suggesting significant alterations in both the amplitude and residual plateau of the PB curve. Regression error is systematically higher across large portions of the lesion, reflecting complex, multi-component decay behavior likely associated with keratin production, inflammation, and disrupted epithelial organization. Overall, SCC exhibits the strongest spatial variability among all examined tumor types, potentially reflecting its greater cellular heterogeneity, higher proliferation rate, and more aggressive stromal remodeling compared with BCC.

## 4 Discussion

In this study, we analyzed autofluorescence (AF) photobleaching (PB) kinetics in 84 histologically confirmed non-melanoma skin cancer lesions (53 sporadic BCC, 25 NBCCS-associated BCC, and 6 SCC). The central finding demonstrated by our data is that pixel-wise PB parameter maps (A, *τ*, C) provide spatial information that differs from static AF intensity imaging. Across lesions, AF intensity typically showed reduced fluorescence within the clinically visible tumor region, but PB-derived maps frequently revealed intralesional heterogeneity and, notably, a mismatch between contours suggested by AF intensity and those suggested by A/τ distributions. This observation indicates that dynamic AF decay behavior encodes contrast not captured by steady-state AF intensity alone.

PB behavior is influenced by factors that affect endogenous fluorophore emission and its photochemical environment, including fluorophore composition and binding state, oxygen availability, local microstructure and scattering, cellular density, and metabolic state (17). These established determinants provide a biologically plausible framework for interpreting why PB parameters may vary within and around tumors. In our cohort, sporadic BCCs commonly showed reduced bleaching amplitude (A) and faster decay constants (τ) relative to surrounding skin, often with spatially heterogeneous patterns. Such changes could plausibly arise from altered fluorophore contributions (e.g., metabolic cofactors) and/or differences in tissue organization and optical scattering associated with tumor architecture; however, our study did not include molecular assays or spatial histopathologic registration, and therefore mechanistic assignments remain hypothesis-generating.

NBCCS-associated BCCs often exhibited τ values closer to those of surrounding skin in the representative cases analyzed, despite reduced baseline AF intensity. One plausible explanation is that NBCCS patients tend to be younger than typical sporadic BCC patients, and baseline age-related differences in skin AF and PB kinetics could reduce kinetic contrast between tumor and non-tumor tissue. Importantly, this age-related explanation was not tested directly in the present study (e.g., by age-matched analysis or multivariable modeling) and should be regarded as a working hypothesis for future cohorts designed to evaluate age and other host factors (anatomical site, phototype, background photodamage) as potential confounders or modifiers of PB contrast.

SCC cases showed visually prominent spatial variability across PB parameters in our limited SCC subset. However, given the small SCC cohort (*n* = 6), these observations are presented as exploratory examples and are not intended to establish a reproducible SCC-specific PB signature or support formal comparisons with the larger BCC groups.

The frequently observed mismatch between AF-intensity–defined contours and A/τ-defined regions is a particularly interesting feature of PB mapping, but its biological meaning cannot be inferred from the current dataset. The “extended” kinetic zones could reflect subclinical tumor extension, peritumoral inflammation, actinic/field cancerization changes, stromal remodeling, or keratinization-related effects. Alternatively, they could arise from technical factors such as variable optical coupling, residual motion, surface topography, illumination non-uniformity, melanin/hemoglobin absorption, or fitting/segmentation artifacts. Therefore, interpretations that extended A/τ zones represent deeper or structurally distinct tumor-related alterations remain speculative without validation against ground truth.

From a translational perspective, PB mapping may ultimately complement conventional lesion assessment by providing functional contrast that is partially independent of static AF intensity. Nevertheless, clinical interpretation, particularly claims related to surgical margin assessment, requires careful validation. In the absence of spatially registered histopathological correlation, the clinical relevance of PB-derived boundary mismatch cannot be established, and margin-related implications should be considered a future direction rather than a conclusion of the present work. Prospective studies should prioritize technical validation and reproducibility (standardized acquisition/calibration, robustness to site/phototype/illumination, and model adequacy), quantitative analysis of parameter distributions and heterogeneity, and, critically, orientation-marked excisions with ex vivo imaging and point-by-point registration between imaging-derived contours and histologic tumor boundaries.

This study has several limitations. Most importantly, we did not perform spatially registered histopathological correlation with the PB-derived maps, so the biological meaning of boundary mismatch and “extended” kinetic zones remains unknown and may reflect tumor-related or non-tumoral processes or technical artifacts. Second, the cohort was unbalanced across tumor types, particularly for SCC (*n* = 6), which limits generalizability and precludes statistically meaningful comparisons; SCC-related findings should therefore be interpreted strictly as preliminary, hypothesis-generating observations. Third, the analysis was primarily qualitative and did not evaluate systematic performance metrics (e.g., sensitivity/specificity, ROC analysis). Finally, technical constraints include the use of a single excitation wavelength (405 nm) and 8-bit RGB detection, which limits spectral discrimination and dynamic range; residual motion, surface topography, and variable optical coupling may influence fitted parameters, particularly in low-signal regions or areas affected by specular artifacts. Inter-patient variability (anatomical site, skin phototype, vascularity, keratinization, baseline photodamage) likely contributes to observed patterns and should be explicitly modeled in larger, prospectively designed datasets.

## 5 Conclusion

This work shows that AF PB imaging can reveal distinct kinetic signatures across sporadic BCC, nevoid BCC, and SCC. While all tumor types exhibited reduced baseline AF, the photobleaching-derived maps (A, τ, C) demonstrated qualitative patterns not apparent in steady-state imaging, including extended zones of altered kinetics and intralesional heterogeneity. These findings suggest that PB kinetics are sensitive to biochemical and structural changes that extend beyond visually defined boundaries, offering potential benefits for improved noninvasive characterization and preoperative margin assessment. Because we did not perform spatially registered correlation with histopathology, the biological and clinical significance of the observed boundary mismatches (extended τ/A zones) remains undetermined, and validating these patterns against histologic margins is an essential next step. Further studies with larger cohorts and histological correlation are needed to establish the diagnostic significance of these observations and to determine whether PB mapping can be translated into clinical practice. In particular, substantially larger SCC cohorts will be required to test whether the exploratory SCC patterns observed here are reproducible and clinically meaningful.

## Data Availability

The raw data supporting the conclusions of this article will be made available by the authors, without undue reservation.
